# Percutaneous endoscopic gastrostomy for palliative oncology patients – a safety analysis in a retrospective cohort study

**DOI:** 10.1186/s12904-026-02435-z

**Published:** 2026-11-13

**Authors:** Anselm Pittrof, Tobias Horst Kinzel, Veronika Reich, Leonie Schuhmacher, Christian Bojarski, Andreas Adler, Wilfried Veltzke-Schlieker, Christian Jürgensen, Zainab Al Khatim, Britta Siegmund, Juliane Buchkremer, Federica Branchi, Christian Treese

**Affiliations:** 1 Department of Hematology, Oncology and Cancer Immunology, Charité – Universitätsmedizin Berlin, corporate member of Freie Universität Berlin and Humboldt-Universität zu Berlin, Campus Benjamin Franklin, Hindenburgdamm 30, Berlin, 12203 Germany; 2 Department of Gastroenterology, Infectious Diseases and Rheumatology (including Nutrition Medicine), Charité – Universitätsmedizin Berlin, corporate member of Freie Universität Berlin and Humboldt-Universität zu Berlin, Campus Benjamin Franklin, Berlin, Germany; 3 Department of Hepatology and Gastroenterology, Charité – Universitätsmedizin Berlin, corporate member of Freie Universität Berlin and Humboldt-Universität zu Berlin, Campus Virchow and Mitte, Berlin, Germany; 4 Cluster of Excellence ImmunoPreCept, Charité – Universitätsmedizin Berlin, Berlin, Germany

**Keywords:** Percutaneous endoscopic gastrostomy (PEG), Palliative oncology, Direct puncture technique, Pull-through technique, Gastropexy, Hybrid PEG, Complications, Mortality

## Abstract

**Background:**

Patients with advanced cancer who are receiving palliative care represent a highly vulnerable population. The role of percutaneous endoscopic gastrostomy (PEG) in this setting is controversial, particularly when life expectancy is limited. Our study aimed to assess complication rates following PEG placement in palliative oncology patients, as well as to compare outcomes with a non-palliative cohort. This comparison was made to identify safety signals that might argue against PEG placement in this population. Additionally, we aimed to evaluate the safety profiles of different PEG techniques.

**Methods:**

In this retrospective cohort study, we analyzed patients who underwent PEG placement at the three endoscopic units of Charité – Universitätsmedizin Berlin between 2016 and 2021. Of these patients, 146 had advanced malignancy and were receiving palliative care. These patients were compared with 1.423 patients who underwent PEG placement in a non-palliative setting. Univariate- and multivariate analyses were performed to evaluate peri- and post-interventional complications. We present mortality data with a focus on PEG-related mortality.

**Results:**

Overall complication rates did not differ significantly between palliative and non-palliative patients (37.0% vs. 32.3%; *p* = 0.246), while procedure-related mortality was higher in the palliative cohort (2.1% vs. 0.4% *p* = 0.013), although the number of events was small. In an additional analysis restricted to oncological patients, palliative status was not significantly associated with overall, peri-interventional, minor or major complications in multivariable models adjusted for age, sex, BMI, PEG placement technique, and PEG indication.

**Conclusion:**

PEG placement in patients with advanced cancer who are receiving palliative care is not associated with significantly higher peri- or post-interventional complications compared to patients who are not receiving palliative care. Decisions regarding PEG placement should be made on a case-by-case basis, taking into account the clinical indication, anticipated benefits, patient preferences and procedural risks.

**Supplementary Information:**

The online version contains supplementary material available at https://doi.org/10.1186/s12904-026-02435-z.

## Introduction

Percutaneous endoscopic gastrostomy (PEG) is a widely used intervention for oncology patients receiving palliative care. The primary indications for PEG placement are enteral nutrition and gastrointestinal decompression [1]. Potential benefits include symptom relief, improved nutritional status, enhanced quality of life, and easier care management [2–5].

Regarding enteral nutritional support, oncology patients generally fall into two groups: those with obstructive tumors in the head, neck or upper gastrointestinal region, which are predominantly primary malignancies, and those with metastatic tumors [6]. In a retrospective cohort study of 218 oncology patients who underwent PEG, 49% of patients had head and neck tumors, and 22% had gastrointestinal malignancies [3].

Malignant bowel obstruction represents the second most common reason for PEG placement in palliative care. It occurs in 3–15% of patients with advanced cancer [7] and accounts for up to 27% of PEG placements in oncology patients [6]. For these patients, PEG is primarily used for decompression to alleviate distressing gastrointestinal symptoms. PEG can effectively reduce nausea, vomiting, and abdominal distension, with reported symptom relief rates approaching 90% [8, 9].

Despite these potential benefits, PEG placement in cancer patients remains controversial. Several studies have reported high early mortality rates following PEG insertion. These rates are often due to advanced malignancy rather than complications from the procedure itself. Non-procedure-related 30-day mortality rates range from 13% to 18.5% [3, 5, 6]. Consequently, PEG use in the palliative care continues to be the subject of ethical and clinical debate [10, 11]. The American Society for Parenteral and Enteral Nutrition emphasizes the four ethical principles - autonomy, beneficence, nonmaleficence, and justice - in decisions regarding artificial nutrition and hydration. In patients with advanced cancer, particularly a patient-centered communication approach that incorporates shared decision-making is essential [12]. Therefore, robust clinical data are needed to inform patients, caregivers, and clinicians about the potential benefits and risks of PEG placement in this vulnerable population.

There is a lack of comparative studies evaluating the outcomes of PEG placement in palliative versus non-palliative patients. Most of the available data focuses on oncology patients without explicitly differentiating them according to their palliative care status [3, 8, 13]. In these cohorts, the reported rates of minor complications range from 37% to 69%, and the reported rates of major complications range from 9% to 24%. Consequently, evidence regarding the safety and clinical outcomes of PEG placement specifically in palliative care patients remains limited.

We aimed to evaluate the rate of complications following PEG placement in palliative oncology patients and to compare the outcomes with those of a non-palliative cohort. This study provides data to inform decision-making regarding PEG placement for these patients.

## Materials and methods

### Study design and data collection

This study is based on a previously published large cohort of patients who underwent percutaneous endoscopic gastrostomy (PEG) placement between 2016 and 2021 [14, 15]. Data collected included sex, age, body mass index (BMI), underlying disease, primary indication for PEG insertion, PEG placement technique, occurrence of complications, and follow-up data up to 60 days post-procedure. Ethical approval for this study was obtained from the Institutional Review Board of Charité – Universitätsmedizin Berlin, Germany (EA4/036/18).

### Definition of palliative care

For further analysis, all patients in the cohort who were diagnosed with oncological diseases and identified as being in a palliative care setting were selected. A “palliative care setting” was defined as the documented non-curative treatment of an underlying malignancy at the time of PEG placement. This definition was based on the recorded PEG indication and interdisciplinary tumor board decisions. Additional data, including the date of death and the Eastern Cooperative Oncology Group (ECOG) performance status [16], were collected retrospectively for the palliative care subgroup.

### Follow-up of complications and mortality

Data on major and minor complications were collected over a 60-day follow-up period. These complications were classified according to those in previous studies [3, 8, 13]. Major complications were defined as potentially life-threatening events, while minor complications included all other adverse clinical outcomes. We also recorded peri-interventional events, including termination of the procedure due to significant oxygen desaturation (decrease in SpO₂) or hypotension.

Both non-procedure-related and procedure-related mortality data were collected. Procedure-related mortality was defined as any death directly attributable to PEG insertion.

### PEG procedure

All PEG insertions were performed according to our clinic’s standard operative procedure, which aligns with current ESGE guidelines [17, 18]. Written informed consent was obtained at least 24 h before intervention, and patients fasted overnight. Peri-interventional antibiotic prophylaxis was administered 30 min before the procedure. Patients were placed supine and sedated with propofol and/or midazolam. Oxygenation, heart rate, and blood pressure were monitored continuously. After esophagogastroduodenoscopy the stomach was insufflated with CO₂. Following abdominal skin disinfection, the insertion site was identified by endoscopic transillumination, locally anesthetized with lidocaine, and punctured percutaneously.

Three techniques were used. In the pull-through technique, a thread introduced through a puncture cannula was grasped endoscopically and extracted orally. A PEG tube (usually 15 Fr; 20 Fr for decompression) was attached and pulled into the stomach until the internal retention disc contacted the gastric wall. The internal and external fixation plates were tightened for 24 h.

In the direct puncture technique, gastropexy was performed using a Gastropexie Device II (Fresenius-Kabi Deutschland GmbH, Bad Homburg, Germany), with three to four sutures placed around the insertion site. A trocar with a peel-away sheath was inserted under endoscopic visualization. After trocar removal, a balloon-retained feeding tube (usually 15 or 20 Fr) was advanced through the sheath, and the balloon was filled with water. Gastropexy sutures were removed after 10–14 days.

The hybrid technique combined gastropexy, as described above, with subsequent placement of a standard PEG tube using the pull-through technique [15].

### PEG technique selection

The conventional pull-through technique was used as the standard procedure for PEG placement between 2016 and 2018, except in patients in whom this technique was contraindicated or not feasible due to an obstructing lesion, such as severe oesophageal stenosis. In these cases, the direct puncture technique was used, in accordance with ESGE recommendations supporting the use of the direct percutaneous introducer technique when the pull technique is contraindicated [18].

A retrospective analysis of our institutional data revealed a high rate of complications associated with the conventional pull-through technique. In light of these findings and the lower complication rates reported for the direct puncture technique [14], we hypothesised that gastropexy was a key factor contributing to the improved safety profile. Consequently, in 2019, we changed our institutional standard of care and introduced the hybrid PEG technique, which combines the conventional pull-through method with gastropexy. From 2019 onwards, this technique became the standard procedure for all patients undergoing PEG placement at our institution. Subsequent analyses by our group supported the rationale for incorporating gastropexy, demonstrating significantly higher rates of major and minor complications with the conventional method.

### Statistical analysis

Statistical analysis was performed using IBM SPSS Version 29 (Ehningen, Germany).

The palliative oncologic subgroup was summarized using descriptive statistics and compared with the non-palliative cohort. Categorical variables, including complication rates, were compared using the chi-squared test or Fisher’s exact test, as appropriate. Complications were classified as peri-interventional or post-interventional and are presented as absolute and relative frequencies.

Within the palliative cohort, complications were further analyzed according to sex, age group, BMI, ECOG performance status, tumor entity, and PEG placement technique. Comparisons between patients with and without complications were performed using chi-squared or Fisher’s exact tests, as appropriate. A multivariable binary logistic regression model was used to identify factors independently associated with the occurrence of any complication, using the occurrence of any peri- or post-interventional complication as the binary dependent variable.

Overall survival following PEG placement was estimated using the Kaplan–Meier method.

#### Additional analysis in patients with oncological indications (Supplementary Table S2)

To address potential confounding related to differences in the underlying indications for PEG placement, a subgroup analysis was performed comparing palliative and non-palliative patients with oncological indications. Complication rates were compared using Pearson’s chi-squared test. Separate multivariable binary logistic regression models were fitted for overall, peri-interventional, minor and major complications. Each model was adjusted for age, sex, BMI, PEG placement technique, and PEG indication. Non-palliative oncology patients served as the reference group for palliative status. ECOG performance status was not included because it was available only for the palliative subgroup. Patients with missing covariate data were excluded from the respective multivariable models.

### Categorical grouping

To facilitate statistical analysis, patients were grouped into pre-defined categories.

#### Cancer type

Gynecological and gastrointestinal malignancies were merged into a single category because of the anatomical proximity of their primary or metastatic lesions to the gastrointestinal tract. Less common malignancies, such as urological and dermatological cancers, were combined into an additional group. In the multivariable analysis, all tumor categories were compared with head and neck cancers as the reference group.

#### Procedure

For procedural technique analysis, both direct puncture and hybrid techniques were included in the “gastropexy” subgroup as compared to the classic pull-through.

#### ECOG

ECOG performance scores were grouped, corresponding to good or bad performance status, into two categories (ECOG 1–2 and ECOG 3–4) for univariate analyses and were further separated in the multivariable regression model, with ECOG 4 used as the reference category.

#### Age

Age was dichotomized into two groups (< 65 years and ≥ 65 years) for the analysis.

#### Weight

For univariate analysis, patients were categorized into three groups according to BMI: Group 1, BMI < 18.5 kg/m² (underweight); Group 2, BMI 18.5–24.9 kg/m² (normal weight); and Group 3, BMI ≥ 25 kg/m² (overweight). For multivariate analysis, patients with BMI < 18.5 kg/m² were compared with those in the remaining BMI categories.

## Results

### Patient characteristics by palliative status

Of the 1,569 patients who underwent PEG placement, 146 (9.3%) had an oncological diagnosis in a palliative care setting. Table 1 presents a comparison of baseline characteristics between palliative and non-palliative patients.

**Table 1:** Patient Characteristics of the analyzed cohort, divided by palliative status

Patient characteristics		Palliative group (*N* = 146)	Non-palliative group (*N* = 1423)	*p* (two-sided)
Sex, *N* (%)	Women	69 (47.3%)	477 (33.5%)	< 0.001
	Men	77 (52.7%)	946 (66.5%)	
BMI-Group, *N* (%)	< 18.5	23 (21.3%)	116 (11.2%)	0.003
	18.5–24.9	59 (54.6%)	539 (52.0%)	
	>=25	26 (24.1%)	382 (36.8%)	
	Missing	38	386	
Age-Group, *N* (%)	< 65 years	83 (56.8%)	618 (43.4%)	< 0.001
	>=65 years	63 (43.2%)	805 (56.6%)	
Main Diagnosis, *N* (%)	Malignant	146 (100%)	704 (49.5%)	< 0.001
	Neurologic	0 (0%)	621 (43.6%)	
	Other	0 (0%)	98 (6.9%)	
PEG placement indication, *N* (%)	Dysphagia	84 (57.5%)	976 (68.6%)	0.007
	Aspiration	61 (41.8%)	633 (44.5%)	0.531
	Vigilance-Disorder	34 (23.3%)	527 (37.0%)	< 0.001
	Venting	51 (34.9%)	10 (0.7%)	< 0.001
	Cachexia	50 (34.3%)	294 (20.7%)	< 0.001
PEG-insertion technique, *N* (%)	Pull-Through	93 (63.7%)	980 (68.9%)	< 0.001
	Direct Puncture	32 (21.9%)	113 (7.9%)	
	Hybrid	21 (14.4%)	330 (23.2%)	
Complications, *N* (%)	Peri-interventional	27 (18.5%)	248 (17.4%)	0.612
	Post-interventional			
	- Minor Complications	30 (20.5%)	276 (19.4%)	0.753
	- Major Complications	11 (7.5%)	104 (7.3%)	0.930
Mortality *N* (%)	60 days overall mortality	64 (51.2%)	91 (6.3%)	< 0.001
	Procedure-related 60 days mortality	3 (2.1%)	6 (0.4%)	0.013
	Missing	21	0	

Differences between groups were calculated using the chi-square test or, where appropriate, Fisher's exact test. Significant differences (p < 0.05) are printed in bold. Missing data are noted under the respective category. More than one indication was possible

*BMI* body mass index”, “*PEG* percutaneous endoscopic gastrostomy”

The mean age of patients in the palliative cohort was 61.8 years (range 24–91 years; SD 12.8), which was significantly lower than that of the non-palliative cohort (mean age 66.2 years; range 18–102 years; SD 13.4; Student’s t-test, *p* < 0.001).

In the non-palliative cohort, the most common underlying diagnoses were malignant diseases (49.5%) and neurological disorders (43.6%). In the palliative cohort, tumor types were distributed as follows: gynecological and gastrointestinal malignancies in 62 patients (42.5%); head and neck cancers in 45 patients (30.8%); other tumor types in 20 patients (13.7%), and lung cancer in 19 patients (13.0%). ECOG performance status was available for the palliative subgroup: ECOG 1 in 27 patients (18.5%), ECOG 2 in 46 patients (31.5%), ECOG 3 in 57 patients (39.0%), and ECOG 4 in 16 patients (11.0%).

Compared with non-palliative patients, the palliative cohort included more women and showed a cachectic profile. This was reflected by a significantly lower body mass index and a higher frequency of malnutrition as an indication for PEG placement. Consistent with the palliative setting, PEG venting procedures were performed significantly more frequently in this group.

### Complications

There were no significant differences in the overall occurrence of peri- or post-interventional complications between the palliative and non-palliative cohorts. Table 2 summarizes the observed complications and the number of patients affected. Of all the evaluated complications, nausea was the only one that occurred significantly more frequently in the palliative cohort.

**Table 2:** Comparison of observed complications in palliative and non-palliative patients

Complications	Palliative group (*N* = 146)	Non-palliative group (*N* = 1423)	*p*
Peri-interventional complications	27 (18.5%)	248 (17.4%)	0.612
Oxygen desaturation	23 (16.3%)	216 (16.8%)	0.875
Hypotension	20 (13.7%)	192 (15.0%)	0.805
Premature termination of procedure	3 (2.1%)	23 (1.6%)	0.283
Post-interventional complications	30 (20.5%)	279 (19.6%)	0.801
Minor complications	30 (20.5%)	276 (19.4%)	0.753
Local pain	24 (16.4%)	176 (12.4%)	0.167
- Pain medication required	22 (15.2%)	159 (11.2%)	0.158
Wound infection	16 (11.0%)	126 (8.9%)	0.406
Local bleeding	3 (2.1%)	30 (2.1%)	0.961
Leaking from the PEG-insertion site	12 (8.2%)	80 (5.6%)	0.208
Gastro-intestinal pain/discomfort	15 (10.3%)	100 (7.1%)	0.156
Nausea	11 (7.5%)	49 (3.5%)	**0.015**
Intervention necessary			
- Parenteral nutrition	7 (4.8%)	43 (3.0%)	0.249
- Clinical follow-up examination	28 (19.2%)	222 (15.7%)	0.269
- Radiologic follow-up examination	12 (8.2%)	119 (8.4%)	0.943
- Interventional follow-up examination	10 (6.9%)	77 (5.4%)	0.476
Major complications	11 (7.5%)	104 (7.3%)	0.930
Wound healing complication	5 (3.4%)	48 (3.4%)	0.980
Subcutaneous abscess	3 (2.1%)	21 (1.5%)	0.591
Unplanned ICU admission	4 (2.7%)	23 (1.6%)	0.323
Acute abdomen	5 (3.4%)	40 (2.8%)	0.678
Peritonitis	6 (4.1%)	57 (4.0%)	0.958
Pneumo-peritoneum	2 (1.4%)	45 (3.2%)	0.224
Ileus	1 (0.7%)	8 (0.6%)	0.854
Invasive ventilation	3 (2.1%)	21 (1.5%)	0.591
Major bleeding	1 (0.7%)	5 (0.4%)	0.536
PEG malposition or displacement	8 (5.5%)	58 (4.1%)	0.427
All complications	54 (37.0%)	459 (32.3%)	0.246

Complications that occurred during or after PEG insertion. The headings in bold indicate the number of patients who experienced at least one complication in that category, while the complications listed in smaller font indicate the frequency of the complications themselves. Differences in the incidence of complications were calculated using the chi-square test or, where appropriate, the Fisher’s exact test. Significant differences (*p* < 0.05) are shown in bold. Complication data were not available for all patients (missing data < 10%)

“*ICU *Intensive Care Unit.”

### Univariate analysis of peri- and post-interventional complications

To better understand the occurrence of complications in the palliative group, patients were divided into two groups: those who experienced at least one complication and those who did not. Comparative analyses were then performed to identify potential differences between these groups. The variables assessed included sex, age, body mass index, ECOG performance status, underlying tumor entity, and PEG placement technique. Table 3 shows peri- and post-interventional complications.

**Table 3:** Univariate analysis of factors associated with peri- or post-interventional complications in palliative patients

		*N*	Peri-interventional Complications (*N* = 27)	*p*	Post-interventional complications			
					Minor Complications (*N* = 30)	*p*	Major Complications (*N* = 11)	*p*
Sex	Female	69	11 (15.9%)	0.452	17 (24.6%)	0.247	7 (10.1%)	0.258
	Male	77	16 (20.8%)		13 (16.9%)		4 (5.2%)	
Age group	< 65	83	16 (19.3%)	0.779	20 (24.1%)	0.223	6 (7.2%)	0.873
	>=65	63	11 (17.5%)		10 (15.9%)		5 (8%)	
BMI	< 18.5	23	3 (13.0%)	0.249	2 (8.7%)	0.246	0 (0%)	0.293
	18.5–24.9	59	9 (15.3%)		15 (25.4%)		5 (8.5%)	
	>=25	26	8 (30.8%)		6 (23.1%)		1 (3.9%)	
ECOG	1–2	73	6 (8.2%)	**0.001**	18 (24.7%)	0.219	8 (11%)	0.117
	3–4	73	21 (28.8%)		12 (16.4%)		3 (4.1%)	
Tumor entity	Gynecologic & gastrointestinal	62	10 (16.1%)	0.610	14 (22.6%)	0.659	4 (6.5%)	0.740
	Lung	19	5 (26.3%)		4 (21.1%)		1 (5.3%)	
	Head and neck	45	5 (11.1%)		2 (4.4%)		5 (11.1%)	
	Others	20	7 (35%)		10 (50%)		1 (5%)	
PEG-insertion technique	Pull-through	93	22 (23.7%)	**0.033**	24 (25.8%)	**0.037**	9 (9.7%)	0.194
	Gastropexy-based	53	5 (9.4%)		6 (11.3%)		2 (3.8%)	

Differences were calculated using the chi-square test or, where appropriate, Fisher's exact test. Significant differences (*p* < 0.05) are printed in bold. BMI data was missing for 25% of the patients

“*BMI* body mass index”, “*ECOG* Eastern Cooperative Oncology Group performance status”, “*PEG* percutaneous endoscopic gastrostomy”

### Peri-interventional complications

Twenty-seven patients (18.5%) in the palliative group experienced peri-interventional complications. The leading complications were oxygen desaturation (16.3%) and hypotension (13.7%). Due to the retrospective design of the study, data on the severity of complications was unavailable. These complications were more prevalent among patients with a high ECOG-performance status and those who underwent the pull-through technique. These complications were not associated with demographic characteristics, BMI or tumor entity. In the control cohort, 248 patients (17.4%) experienced peri-interventional complications (see Table 2). There was no significant difference in cumulative probability between the two groups (*p* = 0.612).

### Post-interventional complications

#### Minor complications

Minor complications occurred in 30 patients (20.5%) in the palliative group. The leading complications were local pain in 24 patients (16.4%) and infection in 16 patients (11%). These complications were significantly more common in patients who received a PEG with the pull-through technique than in patients who received a gastropexy (24 vs. 6 patients, *p* = 0.037). There were no differences in demographic characteristics, BMI, ECOG, or tumor type. In the non-palliative cohort, 276 patients (19.4%) experienced minor complications (see Table 2). There was no significant difference in cumulative probability between groups (*p* = 0.753).

### Major complications

Eleven patients (7.5%) experienced major complications. All patients with major complications also suffered from a minor complication. The most frequent major complications were tube dislodgement (8 patients, 5.5%), peritonitis (6 patients, 4.1%), wound healing complications (5 patients, 3.4%), and acute abdomen (5 patients; 3.4%). There were no significant differences in major complications according to sex, age, BMI, tumor type, ECOG performance status, or PEG-placement technique. Among the non-palliative cohort, 104 patients (7.3%) experienced major complications (see Table 2), and there was no significant difference in cumulative probability between groups (*p* = 0.930).

### Laboratory biomarkers and complications

Preprocedural hemoglobin, creatinine, urea, and albumin levels were compared between patients with and without complications. No significant differences were observed for any of these biomarkers when all complications were considered collectively. Similarly, no significant associations were found for peri-interventional or minor complications. In patients with major complications, mean urea levels were higher than in patients without major complications (107.82 mg/dl vs. 52.04 mg/dl; *p* = 0.018), whereas differences in hemoglobin, creatinine, and albumin levels were not statistically significant. (Supplementary Table S1).

### Multivariate model predicting complications

**Fig. 1:**
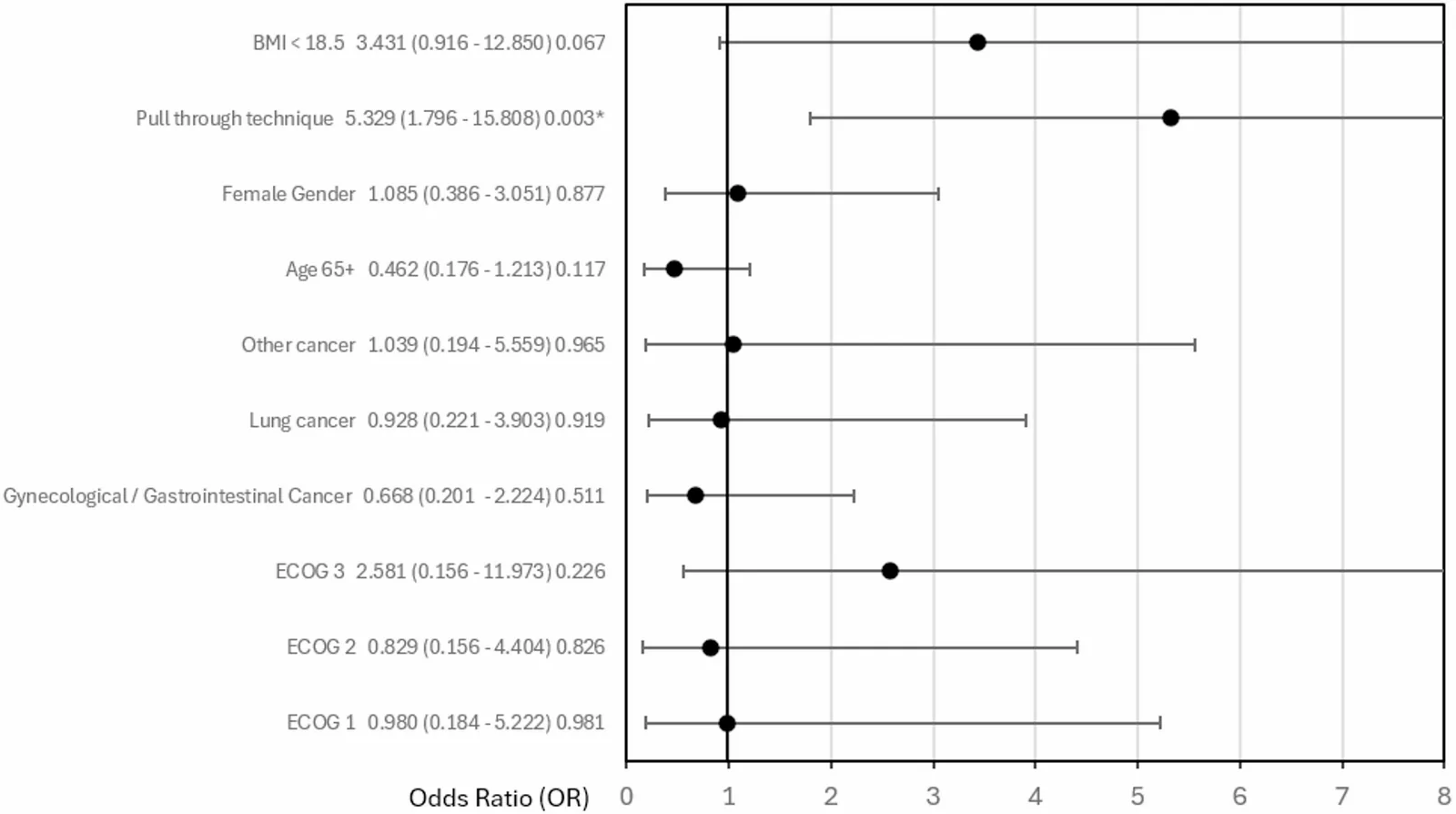
Multivariable logistic regression model predicting the occurrence of a complication. Odds ratios for the occurrence of any type of complication (peri- and post-interventional) for different patient groups. Underweight patients (BMI < 18.5) were compared with normal weighing or overweight patients. The Pull-through technique was compared with gastopexy-based approaches. Cancer types were compared with head and neck tumors as a reference. ECOG groups were compared with ECOG 4 as a reference. Significant differences (p < 0.05) were marked with*. “BMI = Body mass index”, “ECOG = Eastern Cooperative Oncology Group Performance Status”

Binomial logistic regression was performed to predict the influence of sex, age, BMI, tumor type, ECOG performance status, and PEG technique on the probability of a complication occurring (Fig. 1). Of the six variables analyzed only PEG-placement technique significantly predicted the occurrence of a complication (*p* = 0.003). The pull-through technique was associated with a higher likelihood of complications than gastropexy techniques (OR = 5.329, 95% CI [1.791, 15.808]).

Patient cohorts that received different PEG placement techniques were tested for differences in ECOG performance status, age, sex, BMI category and tumor type, where no significant differences were observed.

### Comparison of complications in palliative and non-palliative oncology patients

To address potential confounding due to differences between palliative and non-palliative patients, we performed additional analyses restricted to oncological patients receiving PEG placement. Neither the univariate comparisons nor the multivariable models adjusted for age, sex, BMI, PEG placement technique, and PEG indication demonstrated a statistically significant association between palliative status and overall, peri-interventional, minor, or major complications (Supplement Table S2).

### Mortality

**Fig. 2:**
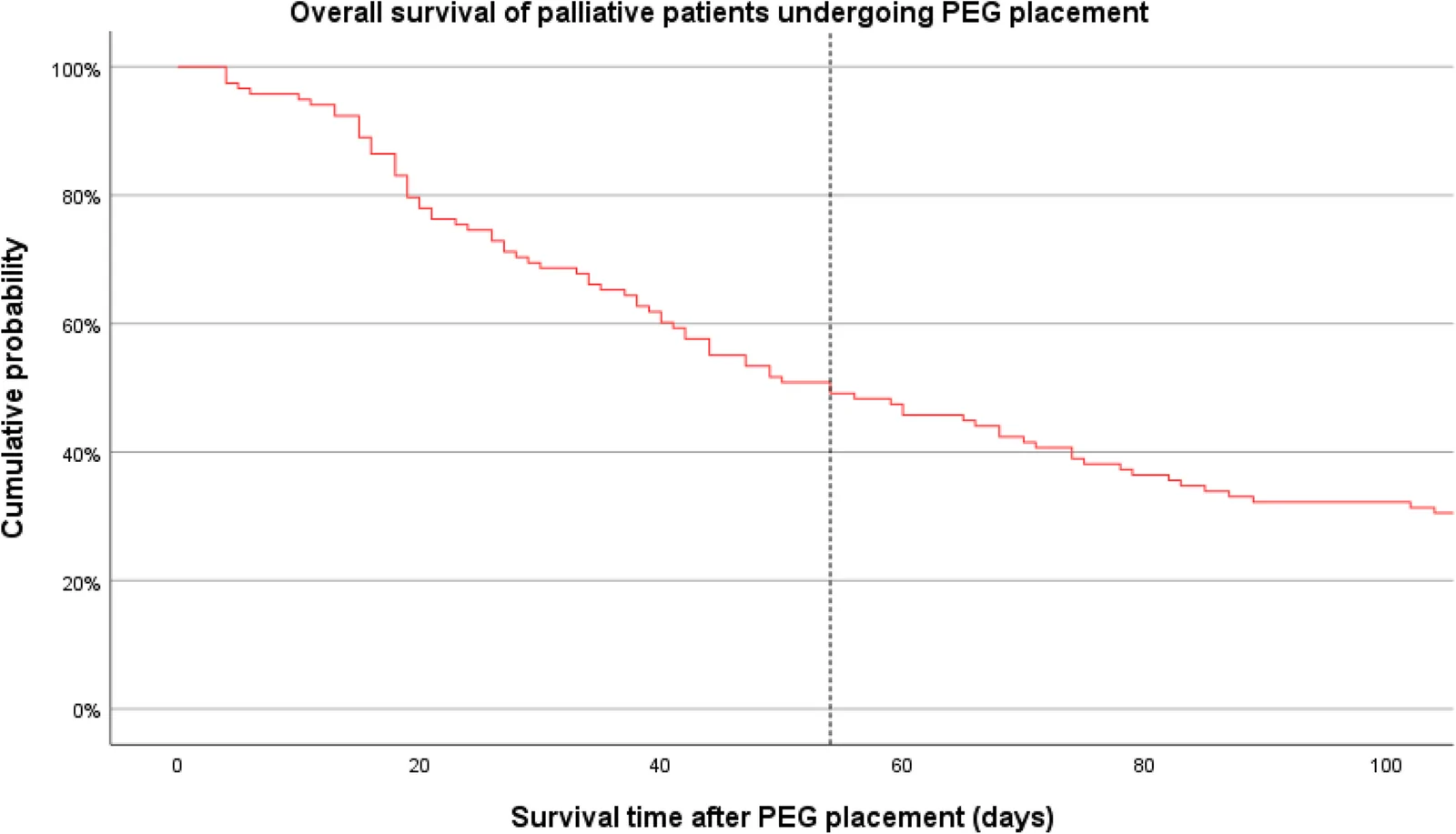
Kaplan-Meier-Survival plot of palliative patients receiving a PEG-placement. Kaplan-Meier-Survival plot of the palliative cohort. The vertical line marks the median overall survival time (54 days beyond PEG placement)

Survival data was available for 118 of the 146 patients. The median overall survival in the palliative cohort was 54 days beyond PEG placement (95% CI: 35.4–72.6) (see Fig. 2).

Although the high overall mortality rate in the palliative care group is primarily due to the advanced stage of the disease, we observed significantly higher 60-day procedure-related mortality in the palliative care group than in the non-palliative care group (3 patients [2.1%] versus 6 patients [0.4%], *p* = 0.013). While procedure-related mortality was higher in the palliative group, the interpretation is limited by the very small number of events.

The three procedure-related deaths were reviewed individually to identify potential contributing factors (see Supplementary S3). In all three cases, the PEG insertion was performed using the pull-through technique. One PEG placement was particularly challenging due to peritoneal carcinomatosis, and the gastropexy was not performed despite the presence of ascites. One patient had a pathogen (Staphylococcus hominis) that was resistant to ceftriaxone, the prophylactic antibiotic administered before the procedure. Escalation of treatment, such as intensive care support or surgical intervention, was not pursued for any of the patients, specifically due to the palliative setting, which may have been contributed to the observed outcome.

## Discussion

### Overall safety of PEG insertion

In this study, we evaluated the complication rates of patients with malignant disease undergoing PEG placement in a palliative setting and compared these outcomes with those of non-palliative patients treated at the same institutions. In the palliative group, the rates of minor and major complication were 20.5% and 7.5%, respectively. These rates were comparable to those in the non-palliative group (19.4% for minor complications, *p* = 0.753; 7.3% for major complications, *p* = 0.930). This major finding shows that, despite being in an overall worse general condition (lower BMI and advanced stage of their disease), patients in the palliative cohort did not experience a significantly higher rate of complications than patients in the non-palliative group. Even when comparing only palliative and non-palliative oncologic patients, no significant differences in complication rates were found, suggesting no significant association between the palliative status and occurrence of complications.

The complication rates in our cohort are consistent with previously published data on gastrostomy placement in oncology patients [3, 13]. A retrospective analysis of 164 patients with advanced cancer who underwent gastrostomy placement revealed an overall complication rate of 55%, despite the patients’ generally better performance status of the patients (45% of them had an ECOG performance status of 1) [13]. In this study, complications were differentiated only into early and late, which limits the possibility of a direct comparison. However, based on our classification system, approximately 48.4% of complications would be considered minor, and 6.6% would be considered major. The high complication rate may be due to the large number of patients with head and neck cancer in the study cohort (77%), which is possibly associated with increased technical difficulty. It may also be due to the fact that 25% of the procedures were performed as percutaneous radiologic gastrostomies.

Similarly, another retrospective cohort study of 218 cancer patients with a high prevalence of head and neck malignancies (49%) and with advanced disease stages (70% stage IV), reported minor and major complication rates of 37% and 9%, respectively. Notably, the rate of minor complications was particularly high compared to our cohort [3].

### Differences in PEG placement technique

We observed a significantly higher rate of peri- and minor post-interventional complications among patients who underwent PEG insertion using the pull-through technique. This held true in the binary logistic multivariate regression model as well, in which the pull-through technique was the only significant predictor of complications. These results align with those from a non-palliative setting, where the direct puncture technique (a gastropexy-based technique) was linked to a 50% decrease in minor complications and an 85.7% decrease in major complications compared to the pull-through technique [15]. We tested for significant differences among patient cohorts receiving different PEG placement techniques, to rule out possible confounders. Clinical parameters such as ECOG or BMI showed no differences, indicating good comparability between the groups.

The existing literature data on the safety profiles of different PEG techniques is heterogeneous. Similarly, other authors reported higher rates of minor complications, predominantly local leakage and infection, with the pull-through technique in a cohort of 423 patients with neurological disorders (50%) and cancer (50%) [19].

Conversely, Kucha et al. compared 598 direct puncture placements with 420 pull placements in 994 cancer patients and reported a higher rate of complications with the direct puncture technique (21.4% vs. 7.1%; HR 2.9 [95% CI 1.9–4.3]; *p* < 0.001). However, this difference was due to higher rates of minor events (15.4% vs. 3.4%), primarily involving detachment of the balloon catheter used in direct PEG puncture. Major adverse events, need for surgical intervention, and 30-day mortality were comparable between the two techniques [20]. Lower rates of infection-related complications, peristomal leakage, and peritonitis have been shown with gastropexy-based PEG techniques, likely due to the avoidance of transoral tube passage and improved gastric fixation have been shown [21]. Therefore, the apparently conflicting findings may be explained by differences in the definition and weighting of complications.

### Biomarkers and complication rates in palliative patients

Due to the retrospective design of the study only hemoglobin, urea, creatinine and albumin were available for our analysis. We found a significant higher urea count in patients suffering from a major complication, whereas differences in hemoglobin, creatinine, and albumin levels were not statistically significant.

Searching the relevant literature, we found two studies, where higher urea count was (non-statistically) associated with major complications ([22]) or shorter survival ([23]). Major Complications in the first cohort were mainly due to major bleeding, which can be partly attributed to uremia. The latter cohort did not differentiate the cause of death, but a generally worse patient condition can be suggested. In our cohort, the main major complications were PEG malposition, peritonitis, acute abdomen and wound healing complications. Although uremia may impair wound healing, a direct pathophysiological relationship between elevated urea levels and the overall spectrum of major complications observed in our cohort remains uncertain. Given the small number of major complications and the exploratory nature of the multiple comparisons, this finding should be interpreted cautiously.

### Overall survival after PEG placement

Overall survival after PEG placement is an important topic of discussion in this study. The median survival time of 54 days in our cohort primarily reflects the progression of underlying malignancy rather than the impact of the PEG in palliative patients. Complication rates were low, while procedure-related mortality was significantly higher than in the control group (3 vs. 6, *p* = 0.013).

Upon closer examination of the procedure-related deaths, major abdominal infection was the ultimate cause of death in all three patients. This finding is consistent with literature data reporting higher infection rates after PEG placement in cancer patients [24]. Moreover, the pull-through technique was used in all three patients, which previous studies have associated with a higher complication rate than gastropexy-based techniques [15, 19]. Finally, an escalation of treatment, which may have improved the outcome, was specifically not pursued due to the palliative setting.

### Limitations

Our study has several limitations: As a retrospective study, the analysis is based on medical records; minor and late complications may have been underreported, and the severity of peri-interventional events could not be assessed. The palliative care status - defined as a documented intention to provide non-curative treatment - may have been misclassified. Clinical benefits and quality of life were not assessed; the data address procedural safety only.

The absence of significant differences in complication rates does not automatically imply comparable safety: the palliative cohort was small, the univariate comparison showed a numerically higher rate of minor complications, and the confidence intervals for major complications were wide (adjusted OR 1.88, 95% CI 0.23–15.7). Multiple comparisons were not adjusted for, so the findings on nausea and urea are exploratory. Procedure-related mortality rested solely on three events and escalation of care was not pursued in these patients.

## Conclusion

In the palliative care setting, treatment decisions are primarily guided by symptom burden, patient preferences, and anticipated quality-of-life outcomes rather than by survival alone. Although quality-of-life outcomes were not evaluated in this study, procedural safety remains a critical factor in PEG placement decisions. In this retrospective cohort, palliative oncology patients did not have statistically significantly higher rates of peri- or post-interventional complications than non-palliative patients. However, the limited sample size, wide confidence intervals, and potential residual confounding preclude conclusions of equivalent safety. Notably, gastropexy-based techniques were associated with lower complication rates in this cohort and may have the potential to reduce procedure-related mortality in this setting. PEG placement in palliative oncology patients should therefore be considered on an individual basis, taking into account the clinical indication, anticipated benefits, patient preferences, and procedural risks.

## Supplementary Information

Supplementary Material 1.

## Data Availability

The data that support the findings of this study are available from the corresponding author, AP, upon reasonable request.
