# Supplementary material for: Percutaneous endoscopic gastrostomy for palliative oncology patients – a safety analysis in a retrospective cohort study
Source: BMC Palliat Care. 2026 Nov 13;25:344. doi: 10.1186/s12904-026-02435-z (PMC13760823; doi:10.1186/s12904-026-02435-z)
Supplement: Supplementary file 1 [file 12904_2026_2435_MOESM1_ESM.docx]

Supplementary PEG-related Mortality

**Supplementary Table 1: Preprocedural laboratory biomarkers according to complication status following PEG placement.**

Values are presented as mean ± standard deviation (SD), with the number of patients available for each parameter. P-values are based on two-sided independent-samples t-tests; Welch’s t-test was used when the assumption of equal variances was violated. All complications comprised peri-interventional, minor, and major complications. P-values were not adjusted for multiple comparisons.

| **Complication** | **Biomarker** | **No complications (n)** | **Mean (+/- Standard Deviation)** | **Complications (n)** | **Mean (+/- Standard Deviation)** | **p-value (2x)** |
| --- | --- | --- | --- | --- | --- | --- |
| All Complications | Hemoglobin (g/dl) | 93 | 10.25 ± 1.77 | 53 | 9.98 ± 1.83 | 0.4 |
|  | Creatinin (mg/dl) | 93 | 0.87 ± 0.53 | 53 | 1.02 ± 0.93 | 0.283 |
|  | Urea (mg/dl) | 90 | 53.77 ± 40.34 | 52 | 60.85 ± 47.01 | 0.365 |
|  | Albumin (g/l) | 77 | 29.97 ± 7.06 | 47 | 29.37 ± 8.48 | 0.686 |
| Periinterventional Complications | Hemoglobin (g/dl) | 119 | 10.24 ± 1.81 | 27 | 9.77 ± 1.67 | 0.22 |
|  | Creatinin (mg/dl) | 119 | 0.87 ± 0.49 | 27 | 1.13 ± 1.26 | 0.306 |
|  | Urea (mg/dl) | 116 | 56.42 ± 41.46 | 26 | 56.08 ± 49.58 | 0.971 |
|  | Albumin (g/l) | 100 | 30.14 ± 7.55 | 24 | 28.07 ± 7.77 | 0.231 |
| Minor Complications | Hemoglobin (g/dl) | 116 | 10.18 ± 1.76 | 30 | 10.05 ± 1.94 | 0.719 |
|  | Creatinin (mg/dl) | 116 | 0.88 ± 0.59 | 30 | 1.07 ± 1.03 | 0.334 |
|  | Urea (mg/dl) | 112 | 52.21 ± 38.26 | 30 | 71.83 ± 54.96 | 0.074 |
|  | Albumin (g/l) | 97 | 29.51 ± 7.08 | 27 | 30.58 ± 9.36 | 0.519 |
| Major Complications | Hemoglobin (g/dl) | 135 | 10.14 ± 1.79 | 11 | 10.27 ± 1.90 | 0.817 |
|  | Creatinin (mg/dl) | 135 | 0.88 ± 0.56 | 11 | 1.47 ± 1.63 | 0.259 |
|  | Urea (mg/dl) | 131 | 52.04 ± 37.69 | 11 | 107.82 ± 65.50 | **0.018** |
|  | Albumin (g/l) | 114 | 29.79 ± 7.34 | 10 | 29.21 ± 10.63 | 0.819 |

**Supplementary Table S2: Univariate comparison and multivariable analysis of complications in palliative and non-palliative patients with oncological indications for PEG placement**

Univariate comparisons were performed using Pearson’s chi-square test. Multivariable binary logistic regression models were adjusted for age, sex, body mass index (BMI), PEG placement technique, and PEG indication. Non-palliative oncology patients were the reference group for palliative status. The adjusted OR therefore represents the odds of the respective complication in palliative compared with non-palliative oncology patients, conditional on the covariates included in the model. The number of patients included in the univariate analyses was 855 for overall and peri-interventional complications and 852 for minor and major complications. The corresponding multivariable models included 852 patients for overall and peri-interventional complications and 849 patients for minor and major complications, owing to missing covariate data. CI, confidence interval; OR, odds ratio; PEG, percutaneous endoscopic gastrostomy.

| **Complication** | **Palliative oncology, n/N (%)** | **Non-palliative oncology, n/N (%)** | **Univariate p-value** | **Adjusted OR (95% CI) for palliative status** | **Adjusted p-value** |
| --- | --- | --- | --- | --- | --- |
| Overall complications | 54/146 (37.0) | 239/709 (33.7) | 0.447 | 0.896 (0.360–2.231) | 0.814 |
| Peri-interventional complications | 27/146 (18.5) | 113/709 (15.9) | 0.337 | 0.600 (0.221–1.626) | 0.315 |
| Minor complications | 30/146 (20.5) | 167/706 (23.7) | 0.418 | 1.295 (0.401–4.184) | 0.665 |
| Major complications | 11/146 (7.5) | 70/706 (9.9) | 0.372 | 1.884 (0.226–15.683) | 0.558 |

**Supplement S3: Case reports of patients with procedure-related mortality**

**Case 1**

A 56-year-old woman with recurrent carcinoma of the base of the tongue and cerebellar metastases presented with severe weight loss due to impaired oral intake. Her condition was further complicated by recurrent infections, including confirmed spondylodiscitis.

A PEG was placed on October 4, 2017, using the pull technique, without any immediate complications. Subsequently, the PEG dislocated. Despite two repositioning attempts, recurrent leakage and local superinfection at the insertion site developed. This led to the PEG removal on November 3, 2017.

Sepsis developed on October 27, 2017, with Staphylococcus hominis and Lactobacillus found in the bloodstream. Despite sequential broad-spectrum antibiotic therapy (ceftriaxone/metronidazole, followed by piperacillin/tazobactam, and subsequently meropenem/vancomycin), there was no clinical improvement. Due to the palliative setting, no surgical intervention was performed for the suspected infectious foci. The patient died on November 7, 2017.

**Case 2**

A 52-year-old man with a recurrent solitary fibrous tumor was receiving palliative outpatient care due to progressive cachexia. A PEG tube was placed on March 8, 2017, using the pull technique, without any immediate complications. Enteral feeding was initiated successfully, and the patient was discharged on March 12, 2017.

On March 15, 2017, the patient presented to the emergency department with abdominal pain, a fever, elevated inflammatory markers, and PEG dysfunction with peritubal leakage. He was diagnosed with four-quadrant peritonitis with sepsis, and intravenous antibiotic therapy with piperacillin/tazobactam was started. However, no pathogen could be identified. In accordance with the patient’s advance directive, surgical intervention was not pursued. He developed septic shock, which led to his death on March 19, 2017.

**Case 3**

On April 18, 2017, a patient with diffuse malignant peritoneal mesothelioma underwent PEG placement, using the pull technique for venting in the setting of peritoneal carcinomatosis and ascites. The procedure was technically challenging due to the patient’s rigid abdominal condition and ascites. The patient developed sepsis following the PEG placement on April 19, 2017. The patient received intravenous antibiotic therapy (piperacillin/tazobactam and metronidazole, later escalated to tigecycline). The pathogens found in the ascites and bloodstream were E. coli, and Enterococcus faecium. Citrobacter freundii was also found in the ascites. A surgical evaluation revealed no PEG dislocation.

On April 25, 2017, the patient developed a secondary Candida sepsis, which did not respond to targeted antifungal therapy (caspofungin). The patient’s condition deteriorated rapidly. Due to the poor overall prognosis, escalation to intensive care was not pursued. The patient died on May 3, 2017.
